# Identification of *SLC22A5* Gene Mutation in a Family with Carnitine Uptake Defect

**DOI:** 10.1155/2015/259627

**Published:** 2015-05-05

**Authors:** Hatice Mutlu-Albayrak, Judit Bene, Mehmet Burhan Oflaz, Tijen Tanyalçın, Hüseyin Çaksen, Bela Melegh

**Affiliations:** ^1^Division of Pediatric Genetics, Department of Pediatrics, Meram Medical Faculty, University of Necmettin Erbakan, Meram, 42080 Konya, Turkey; ^2^Department of Medical Genetics, University of Pécs, Pécs, Hungary; ^3^Szentagothai Research Centre, University of Pécs, Pécs, Hungary; ^4^Division of Pediatric Cardiology, Department of Pediatrics, Meram Medical Faculty, University of Necmettin Erbakan, Meram, 42080 Konya, Turkey; ^5^Tanyalcin Medical Laboratory, Selective Screening and Metabolism Unit, Izmir, Turkey

## Abstract

Primary systemic carnitine deficiency is caused by homozygous or compound heterozygous mutation in the* SLC22A5* gene on chromosome 5q31. The most common presentations are in infancy and early childhood with either metabolic decompensation or cardiac and myopathic manifestations. We report a case of 9-year-old boy with dysmorphic appearance and hypertrophic cardiomyopathy. Tandem MS spectrometry analysis was compatible with carnitine uptake defect (CUD). His sister had died due to sudden infant death at 19 months. His second 4-year-old sister's echocardiographic examination revealed hypertrophic cardiomyopathy, also suffering from easy fatigability. Her tandem MS spectrometry analyses resulted in CUD. We sequenced all the exons of the* SLC22A5* gene encoding the high affinity carnitine transporter OCTN2 in the DNA. And one new mutation (c.1427T>G → p.Leu476Arg) was found in the boy and his sister in homozygous form, leading to the synthesis of an altered protein which causes CUD. The parent's molecular diagnosis supported the carrier status. In order to explore the genetic background of the patient's dysmorphic appearance, an array-CGH analysis was performed that revealed nine copy number variations only. Here we report a novel* SLC22A5* mutation with the novel hallmark of its association with dysmorphologic feature.

## 1. Introduction

Primary systemic carnitine deficiency (PCD) is caused by homozygous or compound heterozygous mutation in the* SLC22A5* gene (MIM # 603377) on chromosome 5q31. PCD is caused by defective activity of the OCTN2 carnitine transporter, resulting in urinary carnitine wasting, low serum carnitine levels, and decreased intracellular carnitine accumulation. Carnitine is a water-soluble quaternary amine that serves as an essential cofactor in transport of long-chain fatty acids across the inner mitochondrial membrane for subsequent beta oxidation. The lack of carnitine (due to OCTN2 transporter deficiency) impairs the ability to use fat as energy source during periods of fasting or stress [1]. OCTN2 transporter deficiency is a lethal, autosomal recessive disorder characterised by early childhood onset cardiomyopathy, with or without weakness and hypotonia, recurrent hypoglycemic hypoketotic seizures and/or coma, failure to thrive, and extremely low plasma and tissue carnitine concentrations (<%5 of normal) [2]. The clinical manifestations of PCD can vary widely with respect to age of onset, organ involvement, and severity of symptoms. The most common presentations are in infancy and early childhood with either metabolic decompensation or cardiac and myopathic manifestations, respectively. Half of the patients typically present in later childhood around the age of 4 years (range: 1 year to 7 years) with dilated cardiomyopathy, hypotonia, muscle weakness, and elevated creatine kinase [3]. Following the finding of low plasma carnitine levels on a screening assay, in a symptomatic individual, or in an asymptomatic at-risk relative, the diagnosis of PCD can be confirmed by* SLC22A5* gene analysis. One molecular genetic testing strategy is sequence analysis of* SLC22A5*. If biallelic pathogenic variants are identified, the diagnosis of PCD is confirmed [4].

## 2. Case Presentation

**Figure 1:**
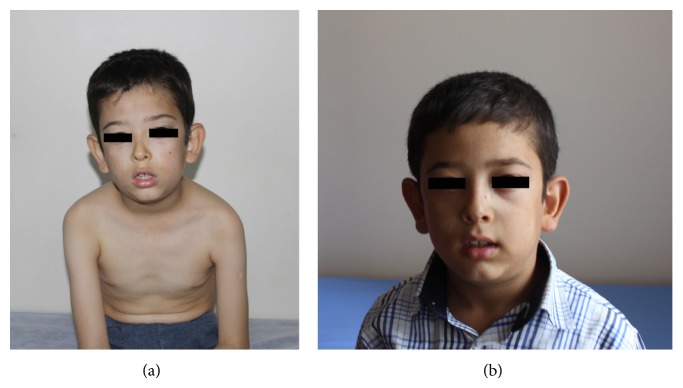
(a) The boy, before carnitine treatment with significant mask face and dysmorphic findings (protruding large ears, hypertelorism, epicanthal folds, swollen eyelids, narrow columella, and small nose). (b) The boy, 2 months after carnitine treatment.

**Figure 2:**
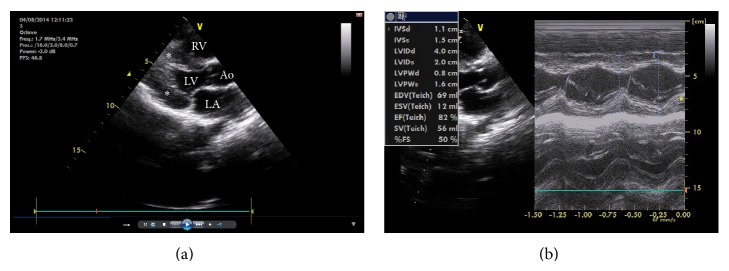
(a) Parasternal long-axis view depicting abnormally small left ventricular end-diastolic cavity, concentric left ventricular hypertrophy (∗), and normal regional wall motion; (b) M-mode tracing across the interventricular septum demonstrating prominent wall hypertrophy. LA: left atrium; LV: left ventricle; RV: right ventricle; Ao: aorta; IVSd: interventricular septum diastolic diameter; IVSs: interventricular systolic diameter; LVIDd: left ventricular end-diastolic diameter; LVISd: left ventricular end systolic diameter; LVPWd: left ventricular posterior wall thicknesses diastolic diameter; LVPWs: left ventricular posterior wall thicknesses systolic diameter; EF: ejection fraction; FS: fractional shortening.

We report a case of 9-year-old boy referred to Pediatric Genetics Clinic because of his dysmorphic appearance and hypertrophic cardiomyopathy. He was suffering from easy fatigability until he started walking. He was the first child of a consanguineous (mother and father were first degree cousins) family. He was born at 35th gestational week, 1500 g and there was not any pre- or postnatal complication. He could hold his head at 3-4 months, sat without being supported at 12th month, and was able to walk at the age of 18 months. On his examination his weight and height were on 50th percentile. His frontooccipital head measurement was below the −2 SD. He had a long, mask face and protruding large ears, hypertelorism, epicanthal folds, swollen eyelids, narrow columella, and small nose (Figure 1(a)). His shoulders were inclined forward while sitting. His muscle strength was 5/5 for four limbs and deep tendon reflexes were depressed. Creatine kinase level was 150 u/L (30–200) and electromyography revealed a myopathic pattern. Tandem MS spectrometry analyses found very low (0.92 uM) free carnitine (>3.8 uM normal) and low (0.13 uM) C3 + C16 acylcarnitines (>2 normal). Carnitine uptake defect (CUD) score was found 244, where 65 or more is interpreted as severe carnitine deficiency. A transthoracic echocardiography was performed revealing abnormally small left ventricular end-diastolic cavity, concentric left ventricular hypertrophy with an ejection fraction of 82%, and normal regional wall motion (Figure 2(a)). The M-mode image shows the increased septal and left ventricular posterior wall thickness more than two standard deviations from the mean (Figure 2(b)). His second sister had died due to sudden infant death at the age of 19 months. His third 4-year-old sister was also suffering from easy fatigability. She was examinated by Pediatric Cardiology Clinic and diagnosed as hypertrophic cardiomyopathy. Her tandem MS spectrometry analyses resulted in CUD. Carnitine supplementation (100 mg/kg/daily) was started orally and both cases urinary carnitine levels increased after treatment. During the follow-up the dosage of carnitine was increased to 200 mg/kg for the boy. No adverse reactions were seen. Both of the siblings showed clinical improvement (Figure 1(b)). CUD was not detected on the mother and fathers' tandem MS spectrometry analysis. Their echocardiographic evaluations were normal. The boy and his sister's molecular genetic test revealed a homozygous* SLC22A5* c.1427 T>G mutation leading to an abnormal protein synthesis; therefore it supported the diagnoses of OCTN2 carnitine transporter deficiency. We sequenced all the 10 exons of the* SLC22A5* gene encoding the high affinity carnitine transporter OCTN2 both in the father's and the mother's DNA using a previously described method [5]. Compared to the reference sequence one mutation (c.1427T>G → p.Leu476Arg) was found in heterozygous form and three already known variants (rs 2631365, rs 274558, and rs 274557) were detected in heterozygous form in the mother's DNA and homozygous form in the father's DNA. The protein coding mutation has not been described so far. Software analysis (PolyPhen-2) predicted that the detected mutation is possibly damaging. Molecular diagnosis supports the carrier status.

An array-CGH analysis was performed on the DNA extracted from whole blood of the proband using the Agilent CytoChip ISCA SurePrint 8 × 60 K oligo-array (Agilent Technologies, USA) to explore the genetic background of the patient's dysmorphic appearance. Nine CNVs (copy number variations) were detected in six chromosomes (Table 1); none of them were proved to be pathogenic based on our in-house database and the publicly available databases such as DECIPHER (Database of Chromosomal Imbalance and Phenotype in Humans using Ensembl Resources) [6], DGV (the Database of Genomic Variants) [7], and Ensembl [8]. Moreover, none of them was found to be associated with dysmorphic feature.

## 3. Discussion

PCD is an autosomal recessive disorder that impairs fatty acid oxidation. It has a frequency of ranging from 1 : 40,000 to 1 : 120,000 newborns in different parts of the world [9–11] and is possibly the second most frequent disorder of fatty acid oxidation after medium chain acyl CoA dehydrogenase deficiency [4]. In the heart, carnitine is essential for normal fatty acid *β*-oxidation and even partial deficiency could lead to organ dysfunction.

Cardiomyopathy is the most common clinical manifestation in children with PCD, which include dilated cardiomyopathy and hypertrophic cardiomyopathy [12]. In patients with PCD dilated cardiomyopathy is more frequently found [13] while cardiac hypertrophy can be seen in heterozygotes for this condition [9]. Heterozygotes for PCD may have mildly reduced plasma carnitine levels [14]. Over time, heterozygotes develop benign cardiac hypertrophy and it is unclear whether they have a higher incidence of cardiomyopathy or heart disease [9, 15]. Newborn screening with tandem mass spectrometry is not routine in our country so our patients are diagnosed late. The cases presented with cardinal symptoms of easy fatigability. Hypertrophic cardiomyopathy was detected by echocardiography. The mother and father who determined heterozygous mutation of* SLC22A5* were screened by tandem mass and no carnitine deficiency was revealed. Their echocardiographic screening was normal as well. No association between genotype and phenotype in PCD was found in previous studies [16]. Patients with identical mutations can have different ages of onset and different types of clinical presentations [17]. Even siblings with the same mutation have different ages of onset and different progressions of disease pointing to the presence of clinical heterogeneity [18]. Affected children, between the ages of 3 months and 2 years, can present episodes of metabolic decompensation triggered by fasting or common illnesses such as upper respiratory tract infection or gastroenteritis. These episodes are characterized clinically by poor feeding, irritability, lethargy, and hepatomegaly. Laboratory evaluations usually reveal hypoketotic hypoglycemia (hypoglycemia with minimal or no ketones in urine), hyperammonemia, and elevated liver transaminases [4]. Both the boy and his elder sister had skeletal and cardiac myopathic signs. However, his little sister died due to sudden infant death. It seems likely that her carnitine deficiency was more severe and she had died with episode of hypoglycemia.

The boy had facial dysmorphia and microcephaly. The chromosomal analysis of this case was normal and this case could not be related to another dysmorphic syndrome. Kilic et al. [18] reported one case CUD with facial dysmorphic findings; however the dysmorphic status of that patient clearly differs from that of our case. Meanwhile, dysmorphic status cannot be explained with known function of carnitine [19–23]. The array-CGH analysis of the proband revealed known benign CNVs only, which are not affected by any genes involved in the carnitine homeostasis and these CNVs are not associated with dysmorphic feature.

More than 60 mutations in the* SLC22A5* gene have been found to cause primary carnitine deficiency. A total of four genetic mutations in the* SLC22A5* gene were identified in this study (Table 2), which of one was described as novel.

Limitation of this study was that the supposed carnitine uptake defect was not confirmed with the investigation of the uptake by fibroblast.

## 4. Conclusion

A new homozygous mutation of c.1427T>G → p.Leu476Arg was identified in these cases. Carnitine uptake defect is one of the rare treatable etiologies of metabolic cardiomyopathies. It should be suspected and searched for by measuring the levels of free and total carnitine in plasma and urine from such patients. The diagnosis of primary systemic carnitine deficiency should be confirmed by identification of biallelic pathogenic variants of* SLC22A5* by molecular genetic testing.

## Figures and Tables

**Table 1:** Detected CNVs, genomic position, and genes concerned based on hg19 (GRCh37).

Chromosome region	Type of variation	Genomic position	Length	OMIM genes involved (OMIM number)
2p21	Duplication	45,172,033–45,172,359	327	*SIX3* (603714)

2q21.1	Duplication	131,280,135–131,280,852	718	*CFC1* (605194)

6p25.3	Deletion	259,911–279,697	19,787	No genes

6q24.2	Duplication	144,328,772–144,328,954	184	*PLAGL1 *(603044)

8p23.1	Deletion	7,187,789–7,410,327	222,540	*DEFB4A* (602215), *DEFB103A* (606611), *SPAG11* (606560)

10q11.22	Deletion	46,550,833–47,776,322	1,225,490	*PTPN20A* (610630), *PTPN20B *(610631), *SYT15* (608081), *GPRIN2 *(611240), *PPYR1 *(601790), *ANXA8 *(602396)

10q11.22	Deletion	48,879,347–49,262,377	383,031	No genes

14q32.33	Duplication	106,405,733–107,209,400	803,668	No genes

Xp22.33	Deletion	77,270–161,183	83,914	No genes

CNV = copy number variation.

**Table 2:** Sequence analysis of the *SLC22A5* gene.

Patient	Gene tested	Genotype
Siblings	*SLC22A5 *	c.285T>C homozygous sequence change (p.Leu95Leu) c.807A>G homozygous sequence change (p.Leu269Leu) c.824+13T>C homozygous sequence change **c.1427 T>G homozygous sequence change (p.Leu476Arg)**
Mother		c.285T>C heterozygous sequence change (p.Leu95Leu) c.807A>G heterozygous sequence change (p.Leu269Leu) c.824+13T>C heterozygous sequence change **c.1427 T>G heterozygous sequence change (p.Leu476Arg)**
Father		c.285T>C homozygous sequence change (p.Leu95Leu) c.807A>G homozygous sequence change (p.Leu269Leu) c.824+13T>C homozygous sequence change **c.1427 T>G heterozygous sequence change (p.Leu476Arg)**
